# Comparing frontal eye field and superior colliculus contributions to covert spatial attention

**DOI:** 10.1038/s41467-018-06042-2

**Published:** 2018-09-03

**Authors:** Anil Bollimunta, Amarender R. Bogadhi, Richard J. Krauzlis

**Affiliations:** Laboratory of Sensorimotor Research, National Eye Institute, Bethesda, MD 20892 USA

## Abstract

The causal roles of the frontal eye fields (FEF) and superior colliculus (SC) in spatial selective attention have not been directly compared. Reversible inactivation is an established method for testing causality but comparing results between FEF and SC is complicated by differences in size and morphology of the two brain regions. Here we exploited the fact that inactivation of FEF and SC also changes the metrics of saccadic eye movements, providing an independent benchmark for the strength of the causal manipulation. Using monkeys trained to covertly perform a visual motion-change detection task, we found that inactivation of either FEF or SC could cause deficits in attention task performance. However, SC-induced attention deficits were found with saccade changes half the size needed to get FEF-induced attention deficits. Thus, performance in visual attention tasks is vulnerable to loss of signals from either structure, but suppression of SC activity has a more devastating effect.

## Abstract (web summary)

Superior colliculus (SC) and frontal eye fields (FEF) contain visuo-motor maps but their contributions to selective attention are not fully understood. Here, the authors perform reversible inactivations of the SC or FEF and report that loss of SC activity has a more devastating effect on attention.

## Introduction

The control of spatial selective attention involves a network of cortical and subcortical brain regions^1,2^. In the cerebral cortex, the frontal eye fields (FEF) have a central role. The FEF was first identified as the primary cortical area involved in the control of saccadic eye movements, based on the ability to evoke saccades with low electrical currents^3^, and subsequent studies described how neuronal activity in the FEF was related to the selection of the saccade target as well as the decision to initiate the saccade itself^4^. A direct link to selective attention was established by studies showing that electrical stimulation of the FEF with currents below the threshold needed to evoke saccades could nonetheless exert effects similar to spatial attention cues, improving visual detection performance in the presence of distractors^5^, and that similar FEF stimulation could influence the activity of visual neurons in extrastriate cortex^6^. These and other findings provided experimental support for the idea that the FEF provides attention-related feedback signals that regulate the quality of sensory processing in the visual cortex^7,8^.

In a similar way, spatial signals in the midbrain superior colliculus (SC) related to saccade preparation^9^ also appear to be involved in target selection and the control of spatial selective attention^10^. Indeed, the first demonstration of neuronal correlates of visual attention were obtained in the SC, in a classic study showing that the visual-evoked activity of SC neurons was larger for stimuli that were subsequently used as the target for a saccade, drawing on the inference that visual attention must shift to the target before the saccade happens^11^. As with the FEF, subthreshold electrical stimulation of the SC changes performance during visual tasks in ways that mimic the allocation of spatial attention, improving the animal’s ability to detect or discriminate visual stimuli presented at retinotopic locations that match site of stimulation in the SC^12,13^. These results support the view of the SC as a priority map important for the control of attention as well as saccades^10,14^, similar to the role proposed for saccade- and attention-related areas in the parietal lobe^15^.

Despite these many studies, the causal contributions of the FEF and SC to selective attention have never been directly compared. In the FEF, reversible inactivation by local microinjection of the chemical agent muscimol, a GABA agonist, increases the reaction time to find targets in the contralateral visual field during a covert visual search task^16,17^. Muscimol inactivation of the FEF also impairs performance accuracy during a covert visual search task that uses valid and invalid spatial cues^18^, although for technical reasons this was done in only one animal. In the SC, reversible inactivation causes large and spatially specific deficits in the ability to discriminate or detect visual stimuli during covert spatial attention tasks^19,20^. Inactivation of the SC also eliminates the improvements in perceptual sensitivity normally made possible by spatial cues during attention tasks^21^. Although these results suggest that both FEF and SC play a causal role in selective attention, the results are not comparable because they used different task designs and visual stimuli as well as different volumes and methods for muscimol injection.

Here, we tested the effects of muscimol inactivation of both the FEF and the SC and took specific steps to provide a fair and direct comparison of their causal contributions. First, we measured the performance of the same monkeys performing the same covert spatial attention task during both sets of reversible inactivations. Second, building on the observation that the effects of inactivation on saccades and visual search co-vary^18^, we adopted the strategy of using the changes in saccade metrics caused by inactivation as a basis for comparing the contribution of each region to selective attention. Our results demonstrate that suppression of activity in either the FEF or SC can cause significant changes in the performance of covert selective attention tasks. However, observing a deficit in the attention task during FEF inactivation required a saccade deficit nearly twice as large as that required during SC inactivation. Thus, assuming that the size of the saccade deficit provides a common benchmark for functional suppression in the two regions, these results indicate that the SC has a larger relative impact on covert spatial attention in our task than the FEF.

## Results

### Inactivation of SC or FEF caused attention task deficits

**Fig. 1:**
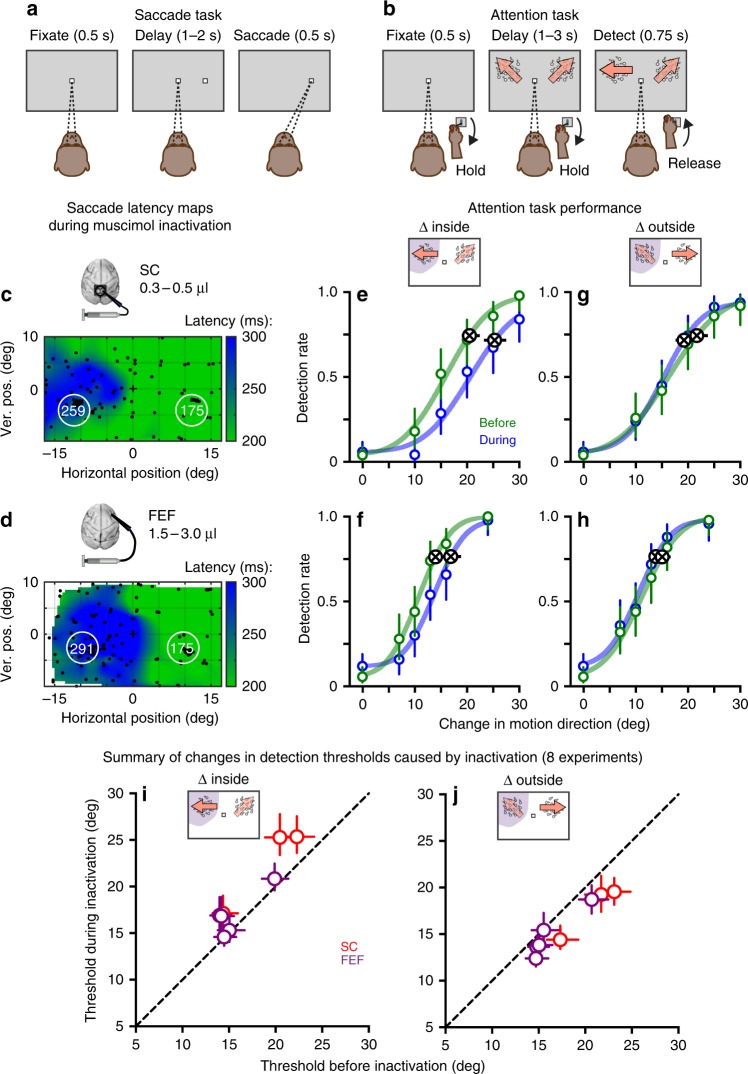
Behavioral tasks and example inactivation results from FEF and SC. **a** Visually guided saccade task used to assess changes in saccade metrics caused by FEF or SC inactivation. Monkeys made a saccade to a peripheral target after a 1–2 s delay. **b** Motion-change detection task used to test how inactivation altered covert spatial attention. During maintained fixation, monkeys released a joystick when the direction of motion changed in either of two motion patches presented in the periphery. **c** Muscimol inactivation of SC caused a localized increase in saccade latencies. Color scale indicates the saccade latencies made to different locations across the visual field, indicated by black dots. White circles indicate the locations of the motion patches in the attention task; white numerals show the average latencies of saccades made to locations that fell within the two patches. **d** Muscimol inactivation of the FEF caused similar increases in saccade latencies. See Supplementary Figure 1 for localization of FEF sites. **e** Psychometric performance before (green) and during (blue) inactivation of SC during one experimental session, when the motion change took place inside the affected portion of the visual field. Colored circles indicate hit rates with 95% CI. Circles with “X” indicate thresholds, defined as the magnitude of stimulus change corresponding to 75% of the signal-driven range in performance—i.e., omitting response bias (lower asymptote) and lapse rate (upper asymptote). Error bars indicate 95% CI. **f** Psychometric performance before and during inactivation of the FEF during one experimental session, when the motion change took place inside the affected portion of the visual field. **g** Psychometric performance before and during SC inactivation from same session as in **e**, when changes occurred outside the affected region. **h** Psychometric performance before and during FEF inactivation from the same session as in **f**, when changes occurred outside the affected region. **i** Summary of eight inactivation experiments in SC (red) and FEF (purple), plotting thresholds during inactivation against thresholds before inactivation, for motion-direction changes inside the affected region of the visual field. Error bars indicate 95% CI. **j** Summary of changes in thresholds when motion-direction changes occurred outside the affected region. The brain images in this figure were originally created by the authors for their *Nature* 2012 doi: 10.1038/nature11497 paper. All rights reserved

Two male macaque monkeys were trained on two tasks: a visually guided delayed saccade task and a covert attention task involving visual change detection. For the saccade task, monkeys made a saccade to a peripheral visual target after the central fixation stimulus was turned off, about 1–2 s after the initial appearance of the peripheral target (Fig. 1a). Across trials, the visual target was placed at various locations in the left or right visual field. For the attention task, monkeys initiated a trial by holding down a joystick, and maintained fixation while two patches of random dot motion were presented simultaneously in the left and right visual field (Fig. 1b). The direction of motion of each dot was drawn from a normal distribution with a mean value (defined as the patch motion direction) and a 16° standard deviation. The monkey was trained to release the joystick if the motion direction changed in either patch, otherwise he should continue to hold down the joystick; the motion-direction change could occur anytime 1–3 s after the onset of the motion stimuli.

Performance on the two tasks was assessed before and during reversible inactivation of the SC or FEF by microinjection of muscimol. During the visually guided saccade task, there were localized increases in latency for saccades directed toward the part of the visual field affected by muscimol injection into the SC (Fig. 1c). These changes in latency provided an independent basis for quantifying the impairment in the parts of the visual field relevant for the motion-change detection task. In this example SC experiment, saccade latencies in the affected patch region were 259 ± 9 ms (mean ± std), whereas latencies in the unaffected patch region were 175 ± 8 ms. There were similar lateralized increases in saccade latency during muscimol injection into the FEF (Fig. 1d and Supplementary Figure 1). In the example FEF experiment, saccade latencies in the affected patch region were 291 ± 9 ms, whereas latencies in the unaffected patch region were again 175 ± 7 ms.

During the covert attention task, in these two example experiments we systematically varied the amplitude of the motion-direction change so that we could construct full psychometric curves and measure detection thresholds. When the motion-direction change occurred in the part of the visual field affected by the muscimol inactivation, the psychometric curves shifted to the right during inactivation of either the SC (Fig. 1e) or the FEF (Fig. 1f), compared to performance before inactivation, indicating that larger changes in motion direction were required to achieve similar levels of detection performance. To quantify these changes in performance, we measured detection thresholds and found that they were significantly increased during inactivation of either the SC (*p* < 0.001) or the FEF (*p* < 0.001), compared to before inactivation. In contrast, when the motion-direction change occurred outside the part of the visual field affected by the muscimol inactivation (Fig. 1g, h), performance improved slightly but significantly (SC before 21.7, during 19.2, *p* < 0.001; FEF before 15.0, during 13.8, *p* < 0.001, Wilcoxon rank-sum test on bootstrap resampled distributions of threshold).

We repeated this experiment several times, obtaining psychometric data before and during inactivation of the SC (*n* = 3) or FEF (*n* = 5), and confirmed the same overall pattern—worse detection performance for changes inside the affected region of the visual field, and better performance for changes outside. For motion-direction changes inside the affected region (Fig. 1i), detection thresholds significantly increased (*p* < 0.001, Wilcoxon rank-sum test on bootstrap resampled distributions of threshold) in 3/3 experiments inactivating the SC, and 4/5 experiments inactivating the FEF (*p* < 0.001). For motion-direction changes outside the affected region (Fig. 1j), detection thresholds significantly decreased in 3/3 SC experiments (*p* < 0.001), and 4/5 FEF experiments (*p* < 0.001). The complementary effects inside versus outside the portion of the visual field affected by muscimol inactivation is consistent with previous reports that inactivation can bias the competition between stimuli that contribute to the perceptual choice, e.g., ref.^19^. Our results demonstrate that reversible inactivation of either the SC or the FEF can cause similar lateralized changes in detection performance, using the same task in the same monkeys.

### Using saccade metrics to compare attention task deficits

We suspected that the variability in the size of these effects on performance across experiments might be due to differences in the efficacy of the inactivation, and to possible differences in the functional roles of the SC and FEF. To get a better handle on this variability, we assembled data from a larger number of inactivation experiments involving these same two tasks but using a single threshold-level change in the detection task, so that many more data sets could be included.

**Fig. 2:**
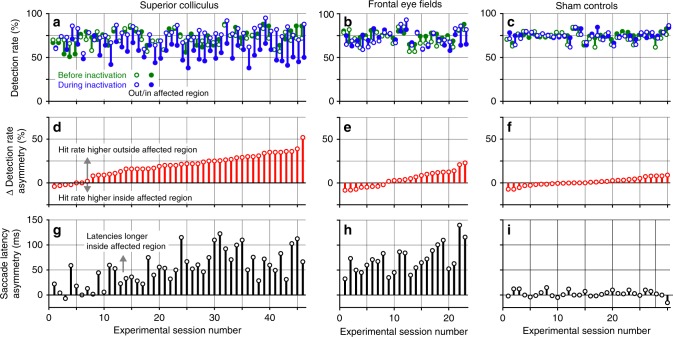
Summary of FEF and SC inactivation effects on attention and saccades. **a** Summary of 46 muscimol inactivations of the SC, showing detection rates before (green) and during (blue) inactivation, when motion-direction changes occurred inside (filled) or outside (open) the affected portion of the visual field. Sessions are rank-ordered based on the size of the change in detection rate asymmetry caused by inactivation. **b** Summary of 23 muscimol inactivations of the FEF, using same conventions as in **a**. **c** Summary of 30 sham control experiments. **d** Changes in detection rate asymmetries caused by SC inactivation. Positive values indicate that, during inactivation, the difference in detection rates between the two patches became more asymmetric in favor of changes outside the affected region. **e** Changes in detection rate asymmetries caused by FEF inactivation. **f** Changes in detection rate asymmetries during sham controls. **g** Asymmetry in the latencies of saccades during SC inactivation observed with the visually guided saccade task. Positive values indicate that saccades made to locations on the affected side had longer latencies than saccades made toward the unaffected side. Only locations falling within the boundary of the two patches in the attention task were considered. Sessions are rank-ordered based on the data in **d**. **h** Asymmetry in the latencies of saccades during FEF inactivation. Sessions rank-ordered based on the data in **e**. **i** Asymmetry in the latencies of saccades during sham controls. Sessions rank-ordered based on the data in **f**. See also Supplementary Figure 2 for plots summarizing the details for each inactivation using the data from Tables 1 and 2

The results from these experiments are summarized graphically in Fig. 2, showing the outcomes from 46 muscimol inactivation experiments conducted in the SC, 23 inactivation experiments in the FEF, plus 30 sham control experiments. Details for each inactivation are provided in Table 1 for the SC and Table 2 for the FEF, and summarized graphically in Supplementary Figure 2. The detection rates from each experiment are shown separately (Fig. 2a–c) for change events inside (filled) and outside (open) the affected region of the visual field, before (green) and during (blue) inactivation. Overall, the blue lines are taller than the green ones, indicating that inactivation caused larger asymmetries in detection performance for changes inside versus outside the affected hemifield, and this effect was most evident for the SC (Fig. 2a) and less evident for FEF (Fig. 2b).

**Table 1:** Summary of muscimol injections performed in the SC

Expt #	Saccade latency asymmetry (ms)	Δ Detection rate asymmetry (%)	Scotoma area (deg^2^)	Stimulus overlap with scotoma (%)	Distance from stimulus center to scotoma center (deg)	Center of scotoma (*x*, *y* deg)	Injection volume (μl)	Subject ID
1	22.7	12.8	19	0	5.3	5.4, 2.6	0.3	M
2	67	22.1	158.4	79.6	2.3	10.4, 2.3	0.4	M
3	44.4	8.9	221.5	71.5	4.1	9.0, 0.1	0.3	M
4	75.5	30.1	221	84.3	5	6.5, 0.9	0.4	M
5	−6.9	−1.9	11	3	4	10.3, −2.4	0.5	M
6	6.1	9.1	81.5	0	9.2	12.0, 5.0	0.4	M
7	17.8	0	15.5	4.4	4.5	9.0, 0.5	0.4	M
8	52.2	21.8	200.7	70.9	7.3	14.1, 1.1	0.5	M
9	53.1	10.8	114.8	59.1	6.6	13.0, 0.4	0.5	M
10	28.3	16.2	165	34.8	2.7	10.7, 0.0	0.4	M
11	46.6	23.8	101.8	62.2	4.1	13.0, −1.4	0.4	M
12	59.8	10.2	46.5	36	5.3	13.9, 0.2	0.5	M
13	13.2	2	49.3	1.7	6.3	12.9, −1.3	0.5	M
14	59.2	−1.7	293.8	96.6	3.7	13.5, 1.1	0.5	M
15	72.1	33.7	272.8	75.9	7	13.5, 2.5	0.5	M
16	70.7	27	237.3	94.8	4.6	13.4, 0.0	0.5	M
17	22	−4.2	17.3	8	12.7	20.1, 3.7	0.5	M
18	4.4	−2.7	14.2	5.6	4.1	10.8, 2.8	0.5	M
19	112.7	38.7	96.3	70.8	10.7	18.0, 4.7	0.4	S
20	66.5	52.2	77.5	11.5	3.5	6.25, 3.0	0.3	S
21	83.1	35.2	55	26.5	4	11.3, 3.3	0.4	S
22	59.6	35.2	178	54	2.2	9.5, 2.8	0.3	S
23	55.8	18.8	201	63.3	4.1	10.9, 5.1	0.3	S
24	1.7	7.9	23.1	12.2	4.5	11.3, −0.3	0.4	S
25	49.2	34.9	111.3	32.9	1.8	6.8, 2.1	0.4	S
26	0.1	0.2	21.1	8.8	3.2	5.6, −0.1	0.4	S
27	22.3	16	25	0	12.6	21.5, 1.7	0.4	S
28	40	17.2	142	44.2	5.2	7.5, 8.1	0.3	S
29	33.3	15.8	115	63	4.8	13.0, 0.6	0.4	S
30	32.3	19.9	100.5	45.3	6.7	4.1, −3.1	0.3	S
31	53.6	19.8	176.8	82.1	3.1	8.8, −1.5	0.4	S
32	36.1	15.8	121	39.9	1.7	7.6, 2.7	0.5	S
33	75	16.2	67.8	77.9	2	10.2, 2.5	0.5	S
34	115	21.4	232.1	65.5	7.6	16.2, −0.4	0.5	S
35	50	20.1	189.4	41.2	7.7	5.6, −5.4	0.4	S
36	100	28.7	95.5	52.2	8.6	14.9, 6.3	0.5	S
37	92.5	26.4	195.2	65.3	9.1	2.6, −6.0	0.5	S
38	110	29.2	163.9	69.2	2	9.8, 1.3	0.5	S
39	110	25.2	175.8	88.7	5.2	12.5, −0.8	0.5	S
40	122.5	25.4	210	91.2	6.8	3.0, 6.1	0.5	S
41	31.3	36.2	81.2	88.5	1.2	8.7, 2.2	0.4	S
42	74.8	25	74.7	90.7	2.3	5.7, 0.6	0.5	S
43	28.5	31	130.3	23.3	7.8	10.5, −5.9	0.4	S
44	50.3	29.8	166.6	44.5	4.2	5.7, 5.0	0.5	S
45	102.8	36.2	140.3	65.5	9.3	17.0, 3.0	0.5	S
46	60.4	22.1	110.5	72.8	2.2	5.6, 1.3	0.5	S

**Fig. 3:**
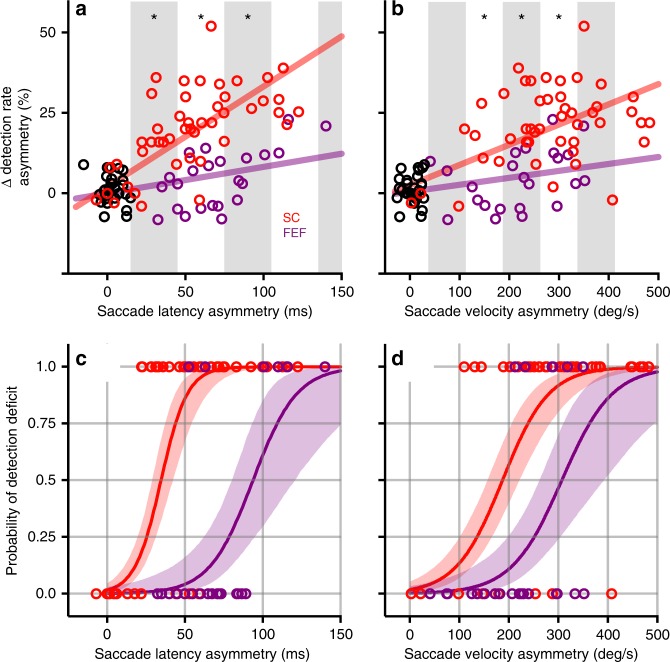
Relating changes in attention task performance to changes in saccade metrics. **a** The change in detection rate is plotted against the saccade latency asymmetry, for each session involving SC inactivation (red), FEF inactivation (purple), or sham control (black). Both the SC and FEF data show a significant linear relationship, but the slope is steeper for the SC data than for the FEF data. Alternating white and gray-shaded regions indicate 30-ms bins in which we compared the changes in hit-rate asymmetry caused by SC and FEF inactivations; asterisks indicate bins for which the values were significantly different (Wilcoxon rank-sum, p < 0.05). **b** The change in detection rate is plotted against the saccade peak velocity asymmetry. Alternating white and gray-shaded regions indicate 75°/s bins in which we compared the changes in hit-rate asymmetry caused by SC and FEF inactivations. Other conventions same as in **a**. **c** The probability of observing a detection deficit is plotted against the saccade latency asymmetry for SC (red) and FEF (purple) inactivation sessions. Each circle shows the result from one experiment involving either the SC or FEF. The smooth curve shows the median, and shaded regions indicate 95% region of the posterior predictive distribution of the *y* variable. **d** The probability of observing a detection deficit is plotted against the saccade peak velocity asymmetry. Same conventions as in **c**. See also Supplementary Figure 3 for direct comparison of the changes in saccade latency and peak velocity caused by inactivation of SC or FEF, and Supplementary Figure 4 for control analyses that address the difference between the numbers of SC and FEF experiments

Experiments are ordered chronologically. Saccade latency asymmetry and Δ detection rate asymmetry are the same values plotted in Figs. 2 and 3. Scotoma area was defined by the areal extent of visual saccade end points with significant increases in latency in each experiment (not fitted or smoothed) and depended on a variety of factors including eccentricity and injection volume; in some cases, the area may be underestimated due to the limited size of our visual display. The stimulus overlap with scotoma was quantified as the percentage of the total stimulus-patch area (on the affected side) that fell within the scotoma. The center of the scotoma was defined as the centroid of the visual saccade end points with significant increases in latency. See also Supplementary Figure 2 for histograms comparing values between SC and FEF

**Table 2:** Summary of muscimol injections performed in the FEF

Expt #	Saccade latency asymmetry (ms)	Δ Detection rate asymmetry (%)	Scotoma area (deg^2^)	Stimulus overlap with scotoma (%)	Distance from stimulus center to scotoma center (deg)	Center of scotoma (*x*, *y* deg)	Injection volume (μl)	Subject ID
1	89.3	10.9	275.7	100	3.8	11.5, −3.8	1.5	M
2	63	14	141.2	62.3	7.1	14.9, 1.3	1.5	M
3	54.9	6.9	115.6	45.2	3.2	6.0, −1.2	1.5	M
4	39.9	4.7	76.3	38.9	4.2	10.8, −1.5	1.5	M
5	32.5	−8.2	95.2	33.5	4	5.0, 4.2	2	M
6	35	1.8	109.2	40.3	6.2	13.9, 5.1	2	M
7	84.4	4	208	61.2	7.2	7.8, −5.5	3	M
8	140	21	259.3	94.8	7.4	10.4, −5.3	3.5	M
9	83.3	−2.2	198.8	76.2	2.8	6.0, 1.5	1.5	S
10	60	−4.6	167.4	66.6	3	10.3, 4.1	1.5	S
11	52.5	12.7	189.4	55	5.8	8.8, 1.2	1.5	S
12	45	−4.8	86.9	77.1	1.3	8.0, 3.3	1.5	S
13	65	8.6	145.7	55.5	4.5	12.2, 2.5	1.5	S
14	72.5	9.8	90	33.2	2.4	6.2, 3.8	1.5	S
15	50	−7.1	202.6	12	8.3	15.6, −1.0	1.6	S
16	67.5	−3.8	65.3	18.4	7	3.0, −1.4	2	S
17	45	2.9	103.2	40.5	1.1	8.8, 0.8	2	S
18	110	12.5	155.6	50.2	5.6	12.7, −1.4	2	S
19	100.8	11.8	238.5	98.2	9.8	17.5, −0.3	2	S
20	71.1	−4	140.7	33.8	5.2	6.1, −3.4	2	S
21	73.4	−8.3	154	58.6	10.1	17.9, 1.3	2	S
22	86.7	3.2	198.3	97.4	6.4	10.4, 2.4	2	S
23	116	23.3	211.5	70.7	8.3	9.8, 4.2	3	S

Same conventions as in Table 1

To condense these effects to a single value for each experiment, we quantified how the asymmetry in detection performance was changed by inactivation:

1 $${\mathrm{\Delta }}{\kern 1pt} {\mathrm{Detection}}\,{\mathrm{rate}}\,{\mathrm{asymmetry}} = ( {{\mathrm{out}}_{{\mathrm{during}}} - {\mathrm{in}}_{{\mathrm{during}}}} ) - ( {{\mathrm{out}}_{{\mathrm{before}}} - {\mathrm{in}}_{{\mathrm{before}}}} )$$

These values report the differences between the heights of the green and blue lines in Fig. 2a–c when the sign of the asymmetry is preserved, or the sum of the heights when the sign of the asymmetry flips during inactivation. The rank-ordered values of the Δ detection rate asymmetry are shown in Fig. 2d–f and were used to define the order of the experimental session values plotted in all of the other panels. These values document the large and consistent changes in detection performance caused by inactivating the SC (Fig. 2d), the smaller effects of inactivating FEF (Fig. 2e), and the seemingly random effects during sham controls (Fig. 2f).

To obtain an independent estimate of the effectiveness of the muscimol inactivations, we next considered the changes in saccade metrics found during the visually guided saccade task during each of these same experimental sessions. We calculated a saccade latency asymmetry, defined as:

2 $${\rm{Saccade}}\,{\rm{latency}}\,{\rm{asymmetry}} = {\rm{latency}}_{{\rm{in}}} - {\rm{latency}}_{{\rm{out}}}$$

These values were positive when the latencies of saccades directed into the affected region of the visual field were longer than those directed outside. We also made similar saccade asymmetry measurements based on saccade peak velocity. The range of the saccade latency asymmetry values (black lines) found during SC inactivation (Fig. 2g) was similar to that found during FEF inactivation (Fig. 2h), indicating that the effectiveness of the muscimol inactivation was comparable across the two sets of experiments. Moreover, sessions with larger changes in detection rate asymmetry also tended to show larger saccade latency asymmetries—the latency asymmetries for the sessions in the right-hand halves of the plots were significantly larger than those in the left-hand halves for both SC (sessions 1–23 versus 24–46, *p* < 0.001 Wilcoxon rank-sum test) and FEF (sessions 1–12 versus 12–23, *p* < 0.05 Wilcoxon rank-sum test).

To more directly assess the relationship between changes in detection rates and saccade latencies caused by muscimol inactivation, we performed linear regression analyses. There was a strong linear relationship between the change in detection rate asymmetry and the saccade latency asymmetry (Fig. 3a) for both the SC (*R*^2^ = 0.83) and the FEF (*R*^2^ = 0.70). However, the slope of this relationship was significantly (*p* < 0.001; Wilcoxon test on bootstrap resampled slope) steeper for the SC (0.31 ± 0.03, standard error, d.o.f = 45) than the FEF (0.08 ± 0.03, d.o.f = 22). Direct comparison of the changes in detection rates within 30-ms saccade latency bins showed significantly larger detection impairments during SC than FEF inactivation, for experiments falling within the mid-range of saccade impairments (regions marked with asterisks in Fig. 3a).

We repeated this analysis using saccade velocity rather than saccade latency and found similar results. There was also a strong linear relationship between the change in detection rate asymmetry and the saccade peak velocity asymmetry (Fig. 3b, see also Supplementary Figure 3) for both the SC (*R*^2^ = 0.78) and the FEF (*R*^2^ = 0.68). Again, the slope of this relationship was significantly (*p* < 0.001) steeper for the SC (0.06 ± 0.01, d.o.f = 45) than the FEF (0.02 ± 0.1, d.o.f = 22), and the detection impairments were larger during SC than FEF inactivation (regions marked with asterisks in Fig. 3b). These results show that, when matched for changes in saccade metrics, inactivation of the SC produces significantly larger changes in performance of the covert attention task than does inactivation of the FEF.

Next, we performed a logistic regression to determine the probability of observing a significant change in performance in the attention task, given the size of the change in saccade metrics caused by muscimol inactivation. For each experimental session, the outcome from the attention task was scored as either a “0” or a “1”, depending on whether the observed change in detection rate asymmetry was significantly different from chance (*p* < 0.001, sign test on bootstrap resampled detection rate asymmetry), and placed along the abscissa at a position defined by either the saccade latency asymmetry (Fig. 3c) or the saccade peak velocity asymmetry (Fig. 3d). We then used the saccade latency or peak velocity asymmetry as a predictive variable in a logistic regression to reproduce the experimentally observed probabilities of obtaining a significant change in detection performance. The resulting logistic fits (with 95% confidence intervals shown by shading) show that SC-induced changes in attention task performance were reliably found with differences in saccade latency of 50–75 ms, whereas FEF-induced changes in performance required latency differences of 100–150 ms (Fig. 3c); similarly, SC-induced changes were found with differences in saccade velocity of 250–350°/s, whereas FEF-induced changes required 350–500°/s (Fig. 3d). Thus, in order to reliably observe an FEF-induced change in performance in the covert attention task, it was necessary for the change in saccade metrics to be 40–100% larger than that required during SC inactivation.

In a set of control analyses, we confirmed that these effects were not simply due to the difference between the number of experiments done in the SC and FEF (Supplementary Figure 4). We randomly subsampled from our SC experiments and repeated (1000 times) the linear regression and probit analyses using the same sample size (*n* = 23) for both the SC and FEF. For the linear regression with saccade latency and peak velocity, we confirmed that the slope for the SC data was almost always significantly greater than that for the FEF data (latency: 999/1000 cases, velocity: 998/1000 cases, Wilcoxon test on bootstrap resampled slope). Similarly, for the probit analysis we confirmed that the midpoint of the function (i.e., the value of *x* at which *y* = 0.5) for the SC data was almost always earlier than that for the FEF data (latency: 999/1000 cases, FEF = x*SC, *x* = 2.4 ± 0.7, mean ± std; velocity: 999/1000 cases, *x* = 1.5 ± 0.3).

Finally, the difference in the effects of SC and FEF inactivation on performance in the attention task cannot be attributed to differences in the scotomas caused by inactivation of the two regions. Comparison of the individual experimental values listed in Tables 1 and 2 shows that there were no significant differences (Wilcoxon rank-sum test) between the SC and FEF experiments in the distances between the centers of the scotoma and the center of the stimulus patch (*p* = 0.64), the percentages of the visual motion stimulus that overlapped with the scotomas (*p* = 0.46), or the overall areas of the scotomas caused by inactivation (*p* = 0.08).

## Discussion

The FEF and SC are the major saccade-related regions of the primate brain that, along with the parietal cortex^8,10,15^, are also implicated in the control of visual spatial attention. Despite the fact that the two structures have very different lineages—the FEF is part of prefrontal cortex, whereas the SC is an evolutionarily ancient midbrain structure—both have been shown to contain oculocentric priority maps with activity that contributes to the orienting of attention as well as to movements of the eyes^14,22^. Why are there both cortical and subcortical priority maps? There is currently no clear answer to this question, although the SC is generally considered to lie closer to the motor system and the FEF closer to voluntary control mechanisms^9,23^. Our study addresses this gap by providing the first data for directly comparing their functional roles. We tested the performance of the same monkeys on the same tasks during muscimol inactivation of the FEF or SC and used the effects of inactivation on saccade metrics as a positive control and independent benchmark for comparing results across the two regions.

Our results demonstrate that both the FEF and SC play causal roles in the control of covert spatial attention. Previous studies of the FEF have used covert visual search tasks and found that reversible inactivation of the FEF impairs the ability to detect the presence of a shape-defined target in the part of the visual field affected by the inactivation^16–18^. The extent of the impairment does not depend on whether the search task is easy or hard^16^, and FEF inactivation does not seem to eliminate the differences in performance caused by presenting subjects with valid versus invalid spatial cues^18^. On the other hand, previous studies of the primate SC have used cued discrimination or detection tasks, and found that focal SC inactivation impairs the ability to judge visual motion stimuli placed in the affected part of the visual field^19,20^; SC inactivation also eliminates the perceptual benefits provided by spatial cues^21^. However, the differences between the task designs and the visual stimuli used in these previous studies of the FEF and SC make it problematic to draw direct comparisons. In contrast, because we studied the two areas using the same tasks, in the same monkeys, our results demonstrate for the first time that inactivation of either the FEF or SC can cause lateralized impairments in the detection of visual motion changes, as evidenced not only by changes in detection rates, but also by similar shifts in psychometric performance curves.

The most surprising aspect of our results is that, when matched for changes in saccades, inactivation of the SC produced much larger changes in attention task performance than inactivation of the FEF. Why were the SC-induced effects on attention larger? One important factor to consider is our decision to use visually guided saccades to document the effects of inactivation on saccades, especially since the FEF seem to be especially important for the control of memory-guided saccades^24,25^. Would we have gotten a different answer if we had used memory-guided saccades? We think the answer is no, for several reasons. First, our use of visually guided saccades did not limit our ability to document saccade deficits during FEF inactivation—they were reproducible, significant, and covered a range comparable to those observed during SC inactivation. The difference we report was not in the extent of the saccade deficits caused by SC and FEF inactivation, but in how these saccade deficits predicted impairments in the attention task. Second, if we had used memory-guided saccades, this would have increased the size of the FEF-induced saccade deficits relative to the SC-induced saccade deficits, while leaving the changes in attention task performance unchanged. As a result, when matched for changes in memory-guided saccades, we would have found an even more extreme version of the same pattern we found with visually guided saccades. Because visually guided saccades depend strongly on activity in both the FEF and SC, they provide a fairer benchmark and positive control. In fact, many would consider the SC to be more important for visually guided saccades because the SC lies closer to the saccade motor output based on its connectivity^26^, the latencies of evoked saccades^27,28^, and the presence of saccade-related activity for all saccades^29^, unlike the FEF;^30^ moreover, inactivation of the SC eliminates the saccades evoked by FEF microstimulation^31^. Thus, our use of visually guided saccades may have tilted the comparison in favor of finding a larger relative attention effect for the FEF, making our findings even more surprising.

On a related point, one could invert the logic of our approach, and treat performance in the attention task as the common benchmark. Applying this alternative framework, we might then conclude that inactivation of the FEF has larger effects on visually guided saccades than does inactivation of the SC, when results are matched for changes in the attention task. This alternative conclusion would be surprising as well because, as mentioned above, previous evidence indicates that the SC lies closer to the saccade motor output. Moreover, this alternative conclusion would also contradict direct evidence showing that FEF lesions produce only temporary deficits in saccades, whereas SC lesions cause permanent impairments^32,33^. Consequently, although we cannot rule out this alternative interpretation, the existing experimental evidence suggests it is much less likely.

Another important concern is that the SC and FEF differ substantially in size and organization, and that these differences might have influenced our results. The SC is smaller (only a few millimeters in width and depth) and comprises a well-organized retinotopic map^34^, whereas the FEF is larger (many millimeters across) and contains a less regular map^3,35^. To compensate for these differences, we injected substantially more muscimol in the FEF (3 μl) than in the SC (0.5 μl), so that the retinotopic extent of the deficits would be comparable. Indeed, our two sets of inactivations produced saccade deficits with the same retinotopic size and the same amount of retinotopic overlap with the visual stimulus used in the attention task (Tables 1 and 2). Thus, despite the structural differences between FEF and SC, we were able to inactivate comparable portions of the retinotopic maps in both brain regions.

A related concern is that our muscimol injections might have affected the saccade-related and attention-related activity in the FEF in a systematically different way from our muscimol injections in the SC. In the SC, neurons located in the intermediate layers with visual and saccade-related activity are the same neurons that are most strongly modulated during selective attention tasks^36,37^. In contrast, in the FEF, the neurons contributing to attention and saccades are not necessarily the same^38^ or even in the same cortical layers—saccade-related neurons are located in the infragranular layers, whereas neurons implicated in visual attention seem to be located in the supragranular layers and are less selective for saccades^39,40^. However, our conclusions do not rely on the assumption that saccades and attention are accomplished by the same neurons, but only that inactivation would be expected to suppress both functions in the same part of the FEF map. Previous studies using similar or smaller injections of muscimol or GABA have reported suppressed activity across a roughly spherical or ellipsoidal volume (elongated along the path of the injection probe) with a radius of ~2 mm or more^41–45^, indicating that our injections would be expected to affect the entire thickness of the FEF. Given that GABA_A_ receptors are located in all layers of cortex^46–48^, if we inhibited saccade-related neurons at a site in the FEF, as documented by changes in saccade metrics, then we almost certainly also inhibited the nearby attention-related neurons. We therefore conclude that the changes in saccade metrics for a particular visual location provide a reasonable positive control for the effectiveness of muscimol-induced suppression of attention-related activity at that same location.

Another important factor is whether our choice of visual stimulus or attention task might have favored one region over the other. The visual stimulus we used—random-dot motion—has been used extensively in studies of both the FEF^49–52^ and the SC^19,21,53–55^, so the particular visual stimulus in our experiments does not favor one area over the other. The use of change detection for the attention task also has precedents in studies of both the FEF^23,56^ and the SC^12,20,37^. Indeed, an important rationale for using a change detection task, rather than a discrimination task, is that this design allowed subjects to report their yes/no choice by holding or releasing a joystick, so that the motor control of the response did not have a spatial component and could therefore be dissociated from the spatial allocation of visual attention. Because the joystick responses for visual changes in the unaffected parts of the visual field showed no deficits, we can rule out a motor explanation for the changes in task performance caused by muscimol inactivation, including the differences between inactivation of the FEF and SC.

Instead, we think the difference between the effects of inactivation in the FEF and SC are due to fundamental differences in the functions of these two brain regions, and that our results illustrate the contrasting susceptibilities of the attention and saccade systems to the temporary loss of signals from the SC and FEF. The FEF provides feedback projections to visual cortical areas, especially area V4, that influence visual processing in a retinotopically selective manner^8^, whereas the SC also exerts a retinotopically selective effect on perceptual choices, but acts downstream from visual cortical areas like MT or MST^20^. The FEF lies in a part of the granular prefrontal cortex that is an evolutionary specialization of primates^57,58^, whereas the SC is present in the brains of all vertebrates, even those without a neocortex^59^. Thus, one possibility is that the FEF is especially important for managing aspects of visual processing related to foveal vision in primates. The selection of visual stimuli from parafoveal or peripheral locations and bringing them to the center of the visual field with saccadic eye movements poses special problems^60,61^, but may also introduce new possibilities for using gaze direction as a part of social communication in primates^62^. In contrast, the SC may contribute to selective attention through mechanisms that predate the evolution of foveal vision, and perhaps lie closer to stages also involved with action selection^63^. If so, then our use of a covert attention task during maintained fixation may not have fully showcased the functional role of the FEF, and other types of attention tasks that require selective shifts of visual processing to the fovea using eye movements rather than hand movements might be expected to produce larger effects.

Nonetheless, in our covert attention task, change detection performance was much more sensitive to disruption of SC activity than FEF activity. Our results therefore provide an informative new constraint on models of covert attention and illustrate the importance of identifying the attention-related circuits and functions linked to the SC and determining how they interact with evolutionarily newer neocortical components.

## Methods

### Animals

Data were collected from two adult monkeys (*Macaca mulatta*) weighing 9–11 kg. All procedures and animal care were approved by the National Eye Institute Animal Care and Use Committee and complied with the Public Health Service Policy on the humane care and use of laboratory animals. Under isoflurane and aseptic conditions, we surgically implanted plastic head-posts and electrophysiology chambers to access the FEF and SC. The FEF chamber was angled 30° lateral of vertical and aimed at a point 18 mm lateral from the midline, 25 mm anterior to the interaural line. The SC chamber was angled 38° to the posterior of vertical and directed at the midline, 15 mm above and 1 mm posterior to the interaural line. Both monkeys were trained on a saccade task and an attention task.

### Saccade task

The saccade task was a delayed visually guided saccade task. Each trial started with the monkeys fixating a small square stimulus (0.25° wide, 50 cd/m^2^) placed at the center of the visual display. After 500 ms of fixation, a second target (0.25° wide, 50 cd/m^2^) was presented at some other location in the visual display. Monkeys were required to maintain fixation until the central fixation stimulus was turned off, 1–2 s after the onset of the peripheral target. At that point, the monkeys should make a saccade to the second, peripheral target and fixate it for at least 500 ms in order to receive a reward. The location of the second target was systematically varied in order to quantify the metrics of saccades to targets across the visual field, as part of the method for documenting the effects of muscimol inactivation on the motor control of saccades.

### Attention task

The attention task consisted of two types of trials: foveal attention (FA) and peripheral attention (PA). The effect of muscimol inactivation on PA trial performance is the focus of the behavioral results reported here; FA trial performance was used as a control to assess attention-related modulation of neuronal activity. In both tasks, monkeys initiated the trial by holding down the joystick and fixating the central fixation spot (0.25°) for the entirety of the trial. During initial fixation, the central spot was surrounded by a color cue (0.35°) that indicated whether the current trial was a PA (red) or an FA (black) trial.

In FA trials, after 500 ms of fixation, two random dot motion stimuli were presented on either side of fixation at 8–10° eccentricity at mirror symmetric locations in the left and right visual hemifield. After a variable delay of 1–3 s, the luminance of the fixation spot decreased on 66% of the trials. Independent of the fixation luminance change, one of the peripheral motion stimulus changed direction after a variable delay of 1–3 s on 66% of the trials. Monkeys should ignore the motion-direction change and instead report the luminance change by releasing the joystick within 300–800 ms to get a fluid reward. If the monkeys released the joystick for a motion-direction change, the trial was aborted. On trials with no luminance change, the monkey was rewarded at the end of the trial for holding the joystick down until the fixation stimulus was turned off. The magnitude of the luminance change was set to be near-threshold level (~75% correct).

In PA trials, after 500 ms of fixation, two random dot motion stimuli were presented at the same stimulus locations as in FA trials. One of the peripheral motion stimuli changed direction after a variable delay of 1–3 s on 66% of the trials; the change could happen in either hemifield with equal probability. Monkeys should report the motion-direction change by releasing the joystick within 300–800 ms to get a fluid reward. Thus, even though the task was structured as a yes/no detection task, the perceptual choice was based on discriminating whether or not the direction of the motion signal changed. On trials with no motion-direction change, the monkey was rewarded at the end of the trial for holding the joystick down until the fixation stimulus was turned off; in these cases, the monkey needed to release and then hold down the joystick again to start the next trial (to prevent a strategy of continuously holding down the joystick). There was no change in luminance of the fixation stimulus in PA trials.

FA and PA trials were run in blocks of 64 trials, interleaved; in each experimental session, we obtained 8–10 blocks of each, half of which were collected before musimol infusion.

### Random dot motion stimulus

The visual motion stimuli were circular patches of moving dots, with the direction of motion of each dot drawn from a normal distribution with a mean value (defined as the patch motion direction) and a 16° standard deviation. The lifetime (10 frames, 100 ms), density (25 dots/deg^2^/s), and speed of the dots (15°/s) were held constant. The radius of the aperture was set to 3°. Luminance of the fixation dot and of each moving dot in the motion patches was 50 cd/m^2^. The background luminance of the monitor was 10 cd/m^2^.

### Electrophysiology

Locations for muscimol injections in the FEF and SC were identified by single-unit recording and electrical microstimulation. Single-unit activity was recorded using tungsten in glass-coated electrodes with impedances of 1–2 MOhm (Alpha Omega Co., Inc., Alpharetta, GA). Electrode position was controlled with a Narishige microdrive. The electrical signal was amplified and recorded online using the OmniPlex system (Plexon Inc., Dallas, TX). Response fields and saccade-related movement fields were mapped by having the monkey perform the visually guided saccade task; we confirmed saccade-related activity consistent with the known activity patterns in the FEF or SC. Electrical microstimulation was also applied (70 ms train duration, 350 Hz, biphasic pulses with duration of 0.25 ms) to evoke saccades with currents (40 μA in FEF; 20 μA in SC) confirming that we were in the FEF or intermediate layers in SC^27,28^. All candidate sites were first identified by neuronal recordings and electrical stimulation prior to the muscimol inactivation experiment; in some cases, the FEF or SC site was verified immediately prior to muscimol injection using the electrode placed within the injection cannula^19^.

### Muscimol infusion

Reversible inactivations of the intermediate layers of the SC (*n* = 46; monkey #1: 18, monkey #2: 28) and FEF (*n* = 23; monkey #1: 8, monkey #2: 15) were done by injecting muscimol (5 mg/ml). The amount of muscimol injected ranged from 0.3 to 0.5 μl in SC and 1.5 to 3.0 μl in FEF. Infusion was done using a custom-made apparatus modified from ref. ^64^, with an injection pump at a rate of 0.5 μl in 10 min.

### Mapping of saccade impairments

We quantified the impairments in saccade metrics by measuring the saccade latency and peak velocity for saccades made to targets at different locations in the visual field, using the visually guided saccade task. Data were collected beginning 30 min after the end of the muscimol infusion, and also at the end of experimental session to assess the spread of muscimol. Results reported in the paper are based on the visually guided saccades obtained 30–45 min after the end of muscimol infusion. To summarize the lateralized impairment in saccades, we computed the asymmetry in saccade latency (and peak velocity), defined as the difference between the average latency (peak velocity) of saccades made into the visual field region subtended by the motion patch in the affected visual field, and those made into the corresponding region in the unaffected visual field, from the data collected after the end of muscimol infusion.

### Psychometric fits and threshold estimation

In separate behavioral sessions prior to muscimol inactivation, psychometric thresholds (75% hit rate) were estimated for each monkey based on performance in the PA motion-direction change detection task using a range of motion-direction changes. The threshold value was then used during the “before” and “during” blocks of task performance during the muscimol inactivation experiments.

In addition, in a smaller number of muscimol inactivation experiments, we used a set of five motion-direction changes during both the “before” and “during” blocks, so that we could construct complete psychometric performance curves. Data from these experiments were fit with a cumulative Gaussian using the *psignifit* toolbox, and included four parameters: mean and standard deviation of the cumulative Gaussian, plus response bias (lower asymptote) and lapse rate (upper asymptote). Threshold was defined as the magnitude of stimulus change corresponding to 75% of the signal-driven range in performance—i.e., 75% of the cumulative Gaussian with the response bias (lower asymptote) and lapse rate (upper asymptote) omitted—so that changes in sensory thresholds during inactivation (reported in Fig. 1) were not confounded by changes in biases or lapses.

### Attention task performance and controls

We summarized the performance during the attention task by computing the asymmetry in hit rate, defined as the hit rate for motion-direction changes in the patch placed in the visual field contralateral to the muscimol injection, minus the hit rate for motion-direction changes in the ipsilateral patch. This hit-rate asymmetry was computed separately for data collected before and during muscimol inactivation for each experimental session, and the change in hit-rate asymmetry was computed by taking the difference in asymmetry before and during inactivation. For monkey #2, a subset of the sessions for the attention task only were performed inside a fMRI scanner while functional imaging data were being collected. For these experimental sessions (SC *n* = 8; FEF *n* = 10), the change in hit-rate asymmetry was computed by taking the difference between the asymmetry on the day of inactivation and the day before inactivation, from data also collected inside the scanner.

In addition, we collected performance on “sham controls” (*n* = 30), which included saline injection sessions (*n* = 8) and no-injection sessions that were matched in experimental timeline to the muscimol injection sessions. Performance during four of the saline “sham controls” were collected inside the scanner.

### Analyses relating attention task performance to saccade metrics

Linear regressions between the changes in hit-rate asymmetry and changes in saccade asymmetry caused by muscimol inactivation were computed using orthogonal linear regression, with saccade asymmetry as the independent variable. Confidence intervals (95%) for slope were computed using Jacknife method. The significance of differences in slopes between SC and FEF were computed by bootstrap resampling. All computations were implemented in Matlab (Mathworks).

We directly compared the changes in hit-rate asymmetry caused by SC and FEF inactivation for experiments with similar saccade deficits. We subdivided the data into equally spaced bins based on the size of the changes in saccade asymmetry (30-ms bins for saccade latency, 75°/s bins for saccade velocity) and determined whether the changes in hit-rate asymmetry were significantly different between the SC and FEF data points falling within each bin (Wilcoxon rank-sum, *p* < 0.05).

Logistic regression was used to estimate the probability of observing a significant change in hit-rate asymmetry during the attention task, as a function of the saccade asymmetry caused by inactivation. To test for significance of the change in hit-rate asymmetry for a given session, we bootstrap resampled (100 samples) all four hit rates (in, out, before, during) to generate a distribution of change in hit-rate asymmetry statistic. Sign test was then used to check for significance (*p* < 0.001 accounting for multiple comparisons). We then used the change in saccade latency or peak velocity as the predictive variable(*x*) in logistic regression- *p*(*y* *=* 1|X,W) = sigm(W^*T*^ *X). Where “*y*” is the categorical variable denoting whether there will be a significant change in hit-rate asymmetry. Laplace approximation was used to estimate the posterior distribution of “W”. Monte Carlo sampling was used to approximate the posterior predictive distribution of “*p*(*y*)”^65^.

### Experimental apparatus

Monkeys viewed the visual stimuli while seated and head fixed in a primate chair (Crist Instrument Inc., Hagerstown, MD, and custom-built) inside a darkened booth. Experiments were controlled using a modified version of PLDAPS^66^. Animals viewed visual stimuli from a distance of 48 cm that were displayed at 1920 × 1200 resolution (~60° × 38°) and 100 Hz frame-rate on a VIEWPixx display (VPixx Technologies, Saint-Bruno, QC, Canada), controlled by a mid-2010 Mac Pro (Apple Inc., Cupertino, CA) running MATLAB (The Mathworks, Natick, MA) with the Psychophysics Toolbox extensions^67–69^. Eye position was recorded using an EyeLink 1000 infrared eye-tracking system (SR Research Ltd., Ottowa, ON, Canada); this system provided the eye position data used to detect saccades and quantify saccade metrics offline using methods described previously^70^. Joystick releases were detected by the onset of the step change in voltage from a Hall effect joystick (CH Products, model HFX-10).

For the subset of attention task sessions that were performed in the scanner, stimuli were back projected onto a screen placed inside the bore of the vertical magnet using an Epson projector controlled by a Windows 2007 machine running MATLAB R2012b (The Mathworks) with the Psychophysics Toolbox extensions^67–69^. The timing of the stimuli and events was controlled by a QNX system running QPCS (Courtesy David Sheinberg). Monkey viewed the screen through a mirror placed in front at a 45° angle. The total viewing distance of the screen was 53 cm. Eye movements were acquired and monitored in the scanner to verify that animals maintained fixation using an iView X system (Version 2.4, SensoMotoric Instruments); the eye signal was calibrated at the beginning of each session. Joystick holds and releases were detected by disruption of an optic fiber transmission using a custom-built device.

### Code availability

Custom code written in MATLAB for running the experiments and analyzing the data is available upon request from the authors.

## Electronic supplementary material

Supplementary Information

## Data Availability

The data that support the findings of this study are available from the corresponding author on reasonable request.
