# Supplementary material for: Comparing frontal eye field and superior colliculus contributions to covert spatial attention
Source: Nat Commun. 2018 Sep 3;9:3553. doi: 10.1038/s41467-018-06042-2 (PMC6120922; doi:10.1038/s41467-018-06042-2)
Supplement: Supplementary file 1 — Supplementary Information [file 41467_2018_6042_MOESM1_ESM.pdf]

Comparing frontal eye field and superior colliculus contributions to covert spatial attention

Bollimunta *et al.*

Supplementary Fig 1: Localization of sites within FEF (related to Figure 1D)

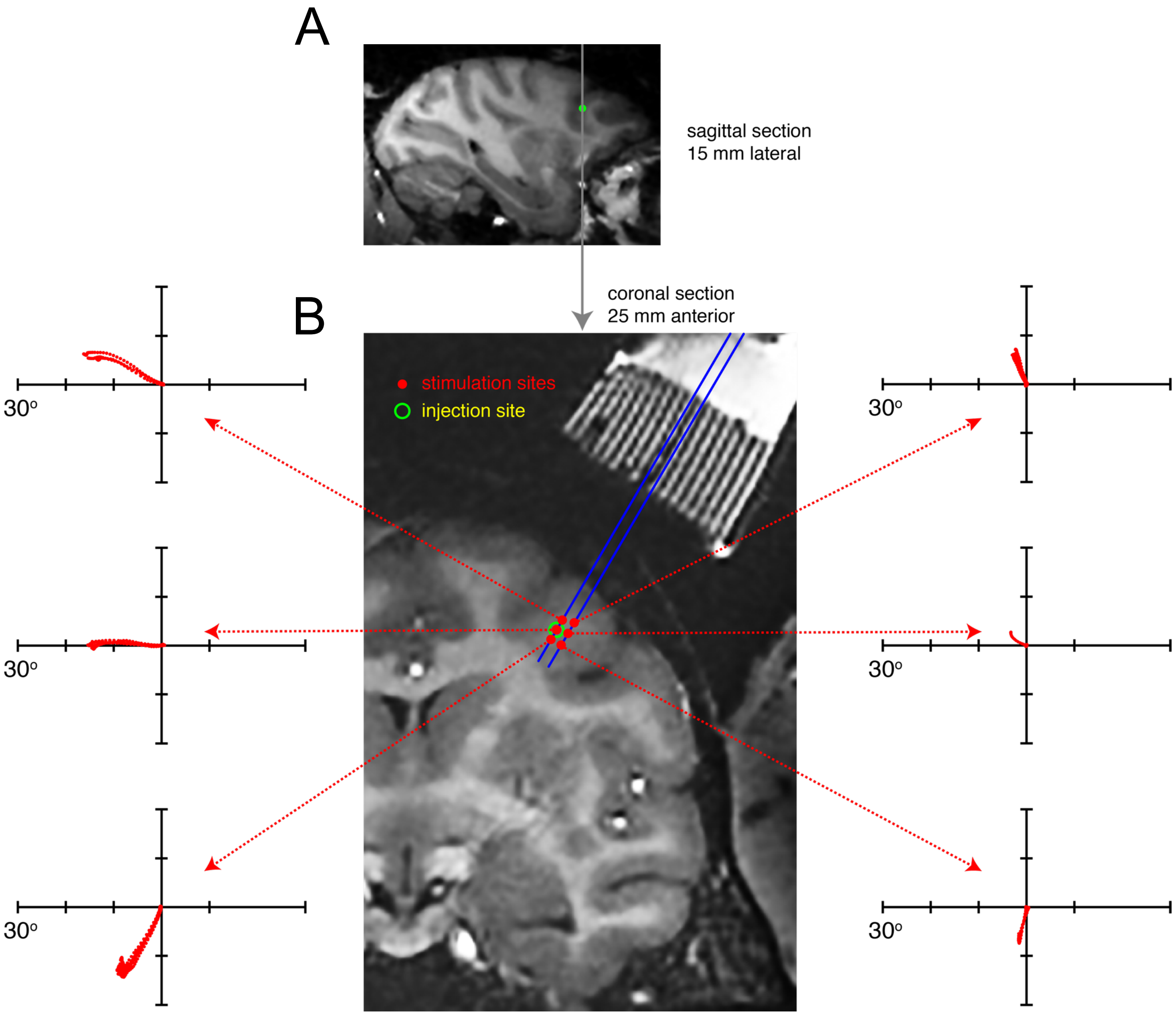

Supplementary Fig 1. Sample stimulation and inactivation sites in the FEF. A) Sagittal section (15 mm lateral) from structural MRI indicating the location of an inactivation site (yellow circle) in the anterior bank of the arcuate sulcus. B) Coronal section (25 mm anterior) taken through this same inactivation site (yellow circle) and illustrating six sample sites (red circles) at which electrical stimulation (70 ms train duration, 350 Hz, biphasic pulses with duration of 0.25 ms, 40 μA) evoked saccadic eye movements with directions and amplitudes (indicated by the trajectories in each of the six corresponding panels) that depended on the location with the FEF. White stripes in upper right of the MRI were produced by the contrast agent placed in the FEF recording grid and the superimposed blue lines indicate the estimated electrode and injectrode paths.

Supplementary Fig 2: Histograms illustrating the similarity between the basic aspects of the inactivation experiments performed in SC and FEF (related to Figure 2 and Tables 1 and 2)

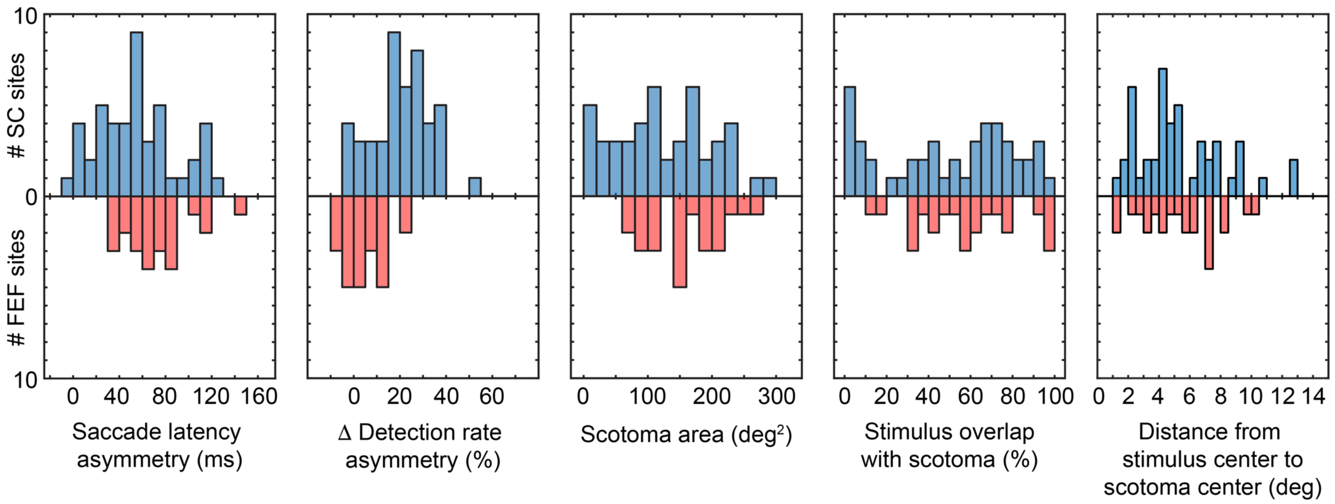

Supplementary Fig 2. Histograms illustrating the similarity between the basic aspects of the inactivation experiments performed in SC and FEF. The histograms compare the distributions of values from SC inactivation experiments (upper row in blue, values from Table 1) with the corresponding distributions of values from FEF inactivation experiments (lower row in red, values from Table 2). The muscimol inactivations in the two areas were well-matched: there were no significant differences (Wilcoxon rank-sum test) between the SC and FEF experiments in the distances between the centers of the scotoma and the center of the stimulus patch (p=0.64), the percentages of the visual motion stimulus that overlapped with the scotomas (p=0.46), or the overall areas of the scotomas caused by inactivation (p=0.08).

Supplementary Fig 3: Comparison of changes in saccade latency and peak velocity caused by inactivation of SC or FEF (related to Figure 3AB)

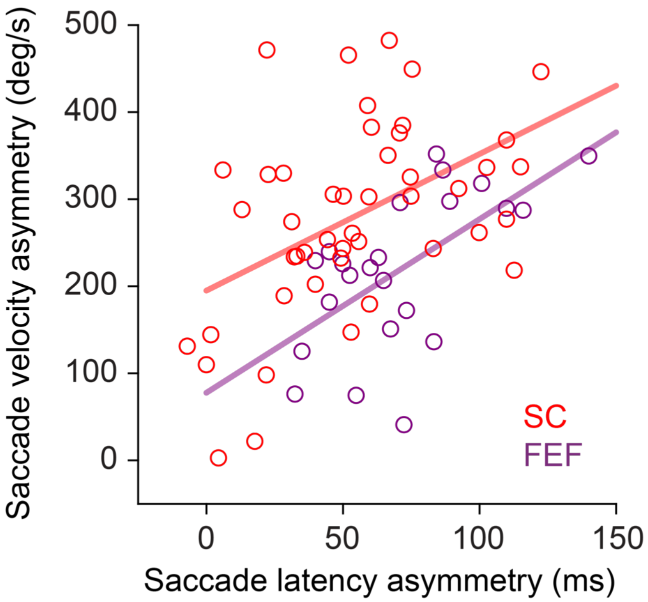

Supplementary Fig 3. Impairments in saccade velocity and latency co-varied across SC and FEF inactivation experiments. The scatterplot directly compares the changes in the two saccade metrics that are plotted separately in Figure 3AB, illustrating that inactivation experiments with larger effects on saccade latency also tended to have larger effects on saccade peak velocity. For both sets of inactivations, there was a significant correlation between the saccade latency asymmetry and the saccade velocity asymmetry (SC: R = 0.476, p = 0.0008; FEF: R = 0.609, p = 0.002, Pearson's). The lines indicate the best-fit linear regressions (SC: slope = 1.570, intercept = 195.0; FEF: slope = 1.996, intercept = 77.9).

Supplementary Fig 4: Control analyses that address the difference between the numbers of SC and FEF experiments (related to Figure 3AB)

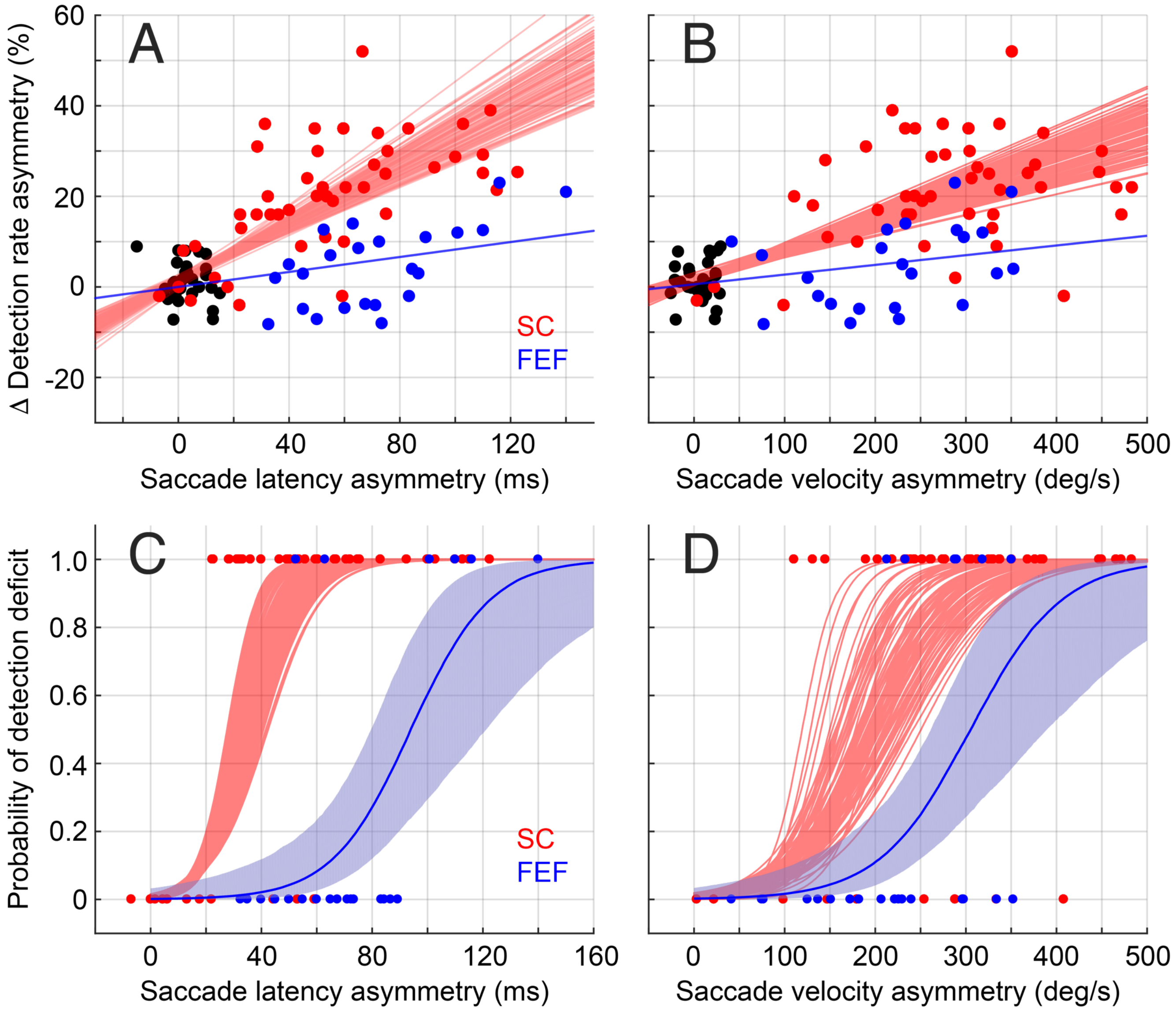

Supplementary Fig 4. Control analyses that confirm that the differences observed between SC and FEF inactivations were not simply due to having done more experiments in the SC (n=46) than the FEF (n=23). We randomly sub-sampled from our SC experiments and repeated (1000 times) the linear regression and probit analyses using the same sample size (n=23) for both the SC and FEF. Each plot shows 100 of the 1000 results, selected randomly. For linear regressions using latency (A) and peak velocity (B), the slope for the SC data was almost always significantly greater than that for the FEF data (latency: 999/1000 cases, velocity: 998/1000 cases, Wilcoxon test on bootstrap resampled slope). For the probit

analyses using latency (C) and peak velocity (D), the midpoint of the function (i.e., the value of x at which y = 0.5) for the SC data was almost always earlier than that for the FEF data (latency: 999/1000 cases, FEF=x*SC, $x = 2.4 \pm 0.7$, mean$\pm$std; velocity: 999/1000 cases, $x = 1.5 \pm 0.3$).
